# Effect of guided imagery on anxiety among pregnant working women

**DOI:** 10.6026/973206300223442

**Published:** 2026-06-30

**Authors:** Patel Jaimikaben Babubhai1, Vankar G.K.

**Affiliations:** 1 Department of Psychiatry Nursing, Parul University, Vadodara, Gujarat, India; 2 Department of Psychiatry, Parul Institute of Medical Science & Research, Parul Sevashram Hospital, Parul University, Vadodara, Gujarat, India

**Keywords:** Guided imagery, anxiety, pregnant women, working women, antenatal mental health, relaxation therapy

## Abstract

Anxiety during pregnancy is a significant maternal mental health problem among working women due to occupational stress and pregnancy- related physiological changes. Therefore, it is of interest to assess the effectiveness of guided imagery in reducing anxiety among pregnant working women in Vadodara, Gujarat. Hence, a pre-experimental one-group pre-test and post-test design was used among 50 participants and anxiety was measured using the Generalized Anxiety Disorder Scale (GAD-7). The mean anxiety score decreased significantly from 24.36 to 16.08 after the intervention (p < 0.001). Thus, we show that guided imagery may serve as a simple and effective non-pharmacological intervention to reduce anxiety during pregnancy.

## Background:

Pregnancy is a complex physiological and psychological process that significantly influences maternal mental health, with increased vulnerability to anxiety during the antenatal period [1, 2, 3]. Although pregnancy is often associated with positive expectations, many women experience emotional challenges due to hormonal fluctuations, physical discomfort and psychosocial adjustments [1, 2]. Antenatal anxiety has emerged as a major global public health concern, with prevalence rates ranging from 13% to over 30% across different populations [1, 2, 3, 4]. Maternal anxiety during pregnancy has been associated with adverse outcomes such as preterm birth, low birth weight and impaired maternal-infant bonding [1, 2, 3]. Elevated stress hormones such as cortisol can affect placental functioning and fetal development, which may lead to long-term emotional and behavioral consequences in offspring [1, 2]. In India, antenatal anxiety remains highly prevalent, with studies reporting that approximately 31% of pregnant women experience moderate to severe anxiety [5]. A recent study conducted in Gujarat (2026) reported that 49% of pregnant women had moderate anxiety and 51% had severe anxiety, indicating a critical need for early intervention [6]. Recent research has identified multiple risk factors contributing to antenatal anxiety, including low socio-economic status, unplanned pregnancy, poor social support and adverse pregnancy history [2, 7]. Qualitative evidence suggests that socio- cultural factors, gender bias and lack of mental health resources influence anxiety among pregnant women, particularly in rural settings [8]. These challenges are further intensified among working women who must balance occupational responsibilities with pregnancy-related demands [2, 4 and 8]. Work-related stress and long working hours significantly contribute to maternal anxiety, especially among women managing dual roles at work and home [4, 8]. Despite increasing awareness, access to safe and effective interventions during pregnancy remains limited, especially in low-resource settings. Pharmacological treatments are often avoided due to potential fetal risks, while psychological therapies may remain inaccessible because of cost, time constraints and stigma [1, 9]. Therefore, there is a growing need for safe, cost-effective and easily implementable non-pharmacological interventions to manage antenatal anxiety [1, 9 and 10]. Guided imagery is a mind-body relaxation technique that promotes psychological relaxation through mental visualization and emotional regulation [10, 11]. Previous studies have demonstrated its effectiveness in reducing anxiety and improving psychological well-being among pregnant women [10, 11]. However, most existing studies have primarily focused on general pregnant populations and have not specifically addressed the unique psychological needs of pregnant working women, who experience additional occupational stress and dual-role responsibilities. Furthermore, although various non-pharmacological interventions have been explored for antenatal anxiety, there is limited evidence specifically evaluating the effectiveness of guided imagery as a targeted relaxation technique in this population. In the Indian context, particularly in regions such as Gujarat where recent studies indicate a high prevalence of antenatal anxiety, there is a lack of structured intervention-based research focusing on safe, cost-effective and easily implementable strategies for anxiety reduction among pregnant working women. Therefore, it is of interest to evaluate guided imagery as a non-pharmacological intervention for reducing antenatal anxiety among pregnant working women and the present study aims to address this gap by assessing its effectiveness in improving anxiety outcomes in this specific population.

## Materials and Methods:

## Study design:

The present study aimed to evaluate the effectiveness of guided imagery in reducing anxiety among pregnant working women in Vadodara, Gujarat. A pre-experimental quantitative one-group pre-test and post-test design was adopted to measure the change in anxiety levels following the intervention. The study was conducted over a period of six months, which included recruitment of participants, administration of the intervention and data collection. The research was carried out in selected urban areas of Vadodara, Gujarat, India. Vadodara is a rapidly developing industrial city with a diverse population of working women employed in government, private and self-employed sectors. The urban setting provided an appropriate environment for studying anxiety among pregnant working women experiencing occupational and pregnancy-related demands.

## Participants and sampling:

The sample for the present study consisted of 50 pregnant working women who were selected using a convenience sampling technique. The sample size was determined based on previous intervention studies involving relaxation techniques during pregnancy, which suggested that a sample of approximately 45 participants would be sufficient to detect a moderate effect size at a significance level of 0.05 with a statistical power of 0.80. To account for potential attrition and ensure adequate sample representation, the final sample size was increased to 50 participants. Participants were included in the study if they were pregnant working women aged between 20 and 40 years, had a gestational age of less than 36 weeks and were willing to participate by providing informed consent. Participants were excluded if they had high-risk pregnancy conditions, a diagnosed psychiatric illness, were unable to participate in the intervention sessions, or declined to participate in the study. These inclusion and exclusion criteria were established to ensure a relatively homogeneous sample and to minimize potential confounding variables that could influence anxiety levels.

## Data collection instruments:

Data for the present study were collected using a self-structured demographic questionnaire and the Generalized Anxiety Disorder Scale (GAD-7). These instruments were used to obtain detailed information regarding the socio-demographic characteristics of participants and to assess their anxiety levels before and after the guided imagery intervention.

## Demographic questionnaire:

A self-structured demographic questionnaire was developed by the researchers to collect information related to socio-demographic, obstetric and occupational characteristics of the participants. The variables included age, educational status, marital status, type of family, monthly family income, gestational age categorized into trimesters, gravida status, type of occupation and working hours per day. The data obtained through this questionnaire were used to describe participant characteristics and to analyze the association between selected variables and post-intervention anxiety levels.

## Generalized anxiety disorder scale (GAD-7):

Anxiety levels among pregnant working women were assessed using the Generalized Anxiety Disorder Scale (GAD-7), a widely used and validated screening tool developed by Spitzer *et al.* [12]. The instrument consists of seven items measuring the frequency of anxiety symptoms experienced over the past two weeks, with responses rated on a four-point Likert scale ranging from 0 (not at all) to 3 (nearly every day). The total score ranges from 0 to 21, with higher scores indicating greater severity of anxiety. The scale has demonstrated high reliability and validity across diverse populations, including maternal health settings [12, 13].

After obtaining informed consent, baseline data were collected using the demographic questionnaire and GAD-7 scale as part of the pre- test assessment. Participants were then introduced to the guided imagery relaxation technique aimed at reducing anxiety and promoting psychological well-being. The intervention was administered over a period of four weeks, with five sessions per week, totalling 20 sessions and each session lasting approximately 20-30 minutes. The sessions involved relaxation exercises, controlled breathing and visualization of calming mental imagery, which have been shown to effectively reduce anxiety and enhance emotional regulation.

## Facilitator qualifications and training:

The guided imagery sessions were conducted under the supervision of two obstetric healthcare professionals, assisted by trained nurses with experience in antenatal counseling and maternal healthcare. The facilitators had prior experience in providing prenatal education and psychological support to pregnant women. Before the implementation of the intervention, the facilitators received structured training in guided imagery techniques, which included theoretical and practical components related to relaxation therapy, session procedures and participant monitoring. The training program covered the basic principles of guided imagery, effective communication with pregnant women and standardized delivery of the intervention. Practice sessions were conducted to ensure consistency in the intervention process and facilitator competency was assessed to ensure adherence to the protocol. Guided imagery is considered a mind-body relaxation technique in which individuals use mental visualization to create calming and positive sensory experiences. Through guided visualization of peaceful environments such as natural landscapes or soothing imagery, participants can achieve psychological relaxation and emotional balance. The technique is grounded in cognitive-behavioral principles that help regulate the autonomic nervous system, promote parasympathetic activity and reduce anxiety-related physiological responses. During the intervention period, participants attended the guided imagery sessions in small groups of 5-8 women in a quiet and comfortable setting within the healthcare environment. Each session began by ensuring that the participants were seated comfortably, followed by instructions from the guided imagery script delivered through audio recordings. The intervention was non-invasive, safe and did not require any specialized equipment, making it an accessible and practical technique for promoting relaxation among pregnant women.

## Data collection procedure:

Data collection was carried out in three phases: pre-test assessment, intervention phase and post-test assessment. During the pre-test phase, participants were informed about the study objectives and procedures and written informed consent was obtained. Baseline anxiety levels were assessed using the GAD-7 scale. Following this, participants underwent the guided imagery intervention for four weeks. Upon completion of the intervention, post-test assessment was conducted using the same tool to evaluate changes in anxiety levels. The comparison of pre-test and post-test scores was used to determine the effectiveness of the intervention. The study adhered to established ethical guidelines for research involving human participants. Ethical approval was obtained from the Institutional Ethics Committee prior to data collection. Participants were assured of confidentiality and their participation was voluntary with the right to withdraw at any stage without any consequences.

## Statistical analysis:

All data were coded, entered and analyzed using the Statistical Package for the Social Sciences (SPSS) software. Descriptive statistics, including frequency, percentage, mean and standard deviation, were used to summarize demographic characteristics and the distribution of pre-test and post-test anxiety levels among participants. Inferential statistics were applied to evaluate the effectiveness of the guided imagery intervention. A paired t-test was used to compare pre-test and post-test anxiety scores of the same participants. The calculated t-value was compared with the critical value at 49 degrees of freedom (df = 49) at a significance level of 0.05. The Chi-square (χ^2^) test was used to examine the association between post-test anxiety levels and selected demographic variables such as age, education, marital status, family type, monthly income, gestational weeks, gravida, occupation type and working hours per day. For all statistical tests, a p-value < 0.05 was considered statistically significant with a 95% confidence level.

## Results and discussion:

The present study evaluated the effectiveness of guided imagery in reducing anxiety among pregnant working women. The findings demonstrated a statistically significant reduction in anxiety levels following the intervention, indicating the effectiveness of guided imagery as a non-pharmacological approach for managing antenatal anxiety. Table 1 presents the demographic and obstetric characteristics of the 50 pregnant working women who participated in the present study. With regard to age distribution, the majority of participants belonged to the 31-35 years age group (40%), followed by 36-40 years (38%). A smaller proportion of respondents were aged 26-30 years (14%), while 8% were in the 20-25 years age group. In terms of educational status, 40% of the participants were graduates, while 34% had completed post-graduation or higher education. Additionally, 14% had completed 10th class education and 12% had completed 12th class education. Regarding marital status, 48% of the participants were married, while 22% were separated, 22% were divorced and 8% were widowed. The majority of participants (62%) belonged to joint families, whereas 38% lived in nuclear families. With respect to monthly family income, 42% of the participants reported earning less than ₹30,000 per month, 32% had an income between ₹30,001 and ₹50,000, 16% reported an income between ₹50,001 and ₹70,000 and 10% had a monthly income above ₹70,000. In terms of obstetric characteristics, 40% of the participants were in the first trimester, 20% were in the second trimester and 40% were in the third trimester of pregnancy. Regarding gravida status, 62% of the participants were primigravida, while 38% were multigravida. With respect to occupational characteristics, 40% of the participants were employed in government jobs, 40% were working in the private sector and 20% were self-employed. Concerning working hours, 60% of the participants reported working more than 8 hours per day, while 40% worked between 6-8 hours daily, indicating the demanding work schedules experienced by many pregnant working women. These demographic findings suggest that pregnant working women often experience multiple responsibilities, which may contribute to increased psychological stress and anxiety during pregnancy. As shown in Table 2, the majority of participants reported moderate to severe anxiety before the intervention, reflecting the psychological burden associated with pregnancy and occupational responsibilities. Following the four-week guided imagery intervention, a substantial reduction in anxiety levels was observed, with many participants shifting to mild or minimal anxiety categories. The mean anxiety score decreased significantly from 24.36 to 16.08 (p < 0.001), indicating the effectiveness of the intervention. These findings are consistent with previous studies demonstrating the effectiveness of guided imagery and relaxation techniques in reducing anxiety during pregnancy. As presented in Table 3, the effectiveness of the guided imagery intervention was assessed using a paired t-test, which revealed a statistically significant reduction in anxiety levels. The mean anxiety score decreased from 24.36 (SD = 5.12) in the pre-test to 16.08 (SD = 4.37) in the post-test, with a mean difference of 8.28. The calculated t-value of 9.74 was statistically significant (p < 0.001), indicating that the intervention had a strong effect in reducing anxiety among participants. This reduction may be attributed to the relaxation response induced by guided imagery, which helps regulate stress and enhance emotional well-being. Similar findings have been reported in earlier studies where guided imagery significantly improved psychological outcomes among pregnant women. Overall, the findings of the present study demonstrate that guided imagery is an effective non-pharmacological intervention for reducing anxiety among pregnant working women. The consistent reduction in anxiety levels and minimal influence of demographic variables highlight its applicability across diverse populations.

As shown in Table 4, the association between post-test anxiety levels and selected demographic variables was analyzed using the chi-square test. The findings indicated that most variables, including age, education, marital status, type of family, monthly income, gestational age, gravida and occupation type, were not significantly associated with anxiety levels (p > 0.05), suggesting that guided imagery was effective across diverse participant groups. However, working hours per day showed a statistically significant association with anxiety levels (χ^2^ = 9.57, p = 0.023), with participants working more than 8 hours per day experiencing relatively higher anxiety. This highlights the impact of occupational workload on maternal mental health and is supported by previous studies indicating that work-related stress significantly contributes to anxiety among pregnant working women [2, 4 and 8]. The findings of the present study demonstrated a significant reduction in anxiety levels among pregnant working women following the guided imagery intervention, as reflected by the decrease in mean anxiety scores from 24.36 to 16.08 (p < 0.001). This improvement may be attributed to the relaxation response induced by guided imagery, which promotes emotional regulation and reduces physiological stress. These results are consistent with previous studies reporting the effectiveness of guided imagery and other mind-body interventions in reducing anxiety during pregnancy [1, 10, 11]. Furthermore, the lack of significant association between most demographic variables and post- test anxiety levels suggests that the intervention is effective across diverse groups. However, the significant association with working hours highlights the influence of occupational stress on maternal mental health, consistent with existing evidence [2, 4 and 8]. The findings of this study have important implications for maternal mental health care. Anxiety during pregnancy is associated with adverse maternal and fetal outcomes, including preterm birth, low birth weight and impaired neurodevelopment, emphasizing the need for effective and safe management strategies [1, 2, 3]. Guided imagery is a simple, cost-effective and non-pharmacological intervention that can be integrated into antenatal counseling and maternal health programs to support early management of psychological distress [9, 10- 11]. The findings also emphasize the role of occupational factors, particularly long working hours, in influencing maternal anxiety, suggesting the need for supportive workplace policies such as flexible working conditions and stress management programs. Furthermore, incorporating mental health screening into routine antenatal care and utilizing national programs such as the Pradhan Mantri Surakshit Matritva Abhiyan (PMSMA) can enhance access to maternal mental health services. Overall, guided imagery represents a scalable and practical approach for improving maternal psychological well-being across diverse populations. The findings of the present study are strongly supported by recent evidence emphasizing the effectiveness of guided imagery in reducing anxiety through mind-body mechanisms. Guided imagery promotes a relaxation response by activating the parasympathetic nervous system, reducing sympathetic arousal and improving emotional regulation. Recent systematic reviews have demonstrated that guided imagery significantly reduces anxiety across clinical and maternal populations [10]. Furthermore, evidence indicates that non-pharmacological interventions play a crucial role in managing antenatal anxiety, particularly when pharmacological approaches are limited due to fetal safety concerns [1, 9]. These findings are consistent with the present study, which demonstrated a significant reduction in anxiety levels following guided imagery intervention.

## Study strengths and limitations:

The present study has several strengths. It focuses on pregnant working women, a population that has received relatively limited attention in maternal mental health research. The study also utilized a standardized anxiety assessment tool (GAD-7) to measure anxiety levels and implemented a structured guided imagery intervention over four weeks, ensuring consistent exposure to the relaxation technique. However, certain limitations should also be acknowledged. The study employed a pre-experimental one-group pre-test and post-test design without a control group, which limits the ability to establish a definitive causal relationship between the intervention and the observed outcomes. In addition, the sample size was relatively small and limited to a specific urban setting, which may affect the generalizability of the findings. Future research should consider randomized controlled trials with larger and more diverse samples to further evaluate the effectiveness of guided imagery interventions. Long-term follow-up studies are also recommended to examine the sustained impact of guided imagery on maternal mental health and neonatal outcomes. Furthermore, exploring the barriers and facilitators to implementing guided imagery programs in antenatal care settings will be essential for scaling up such interventions within maternal healthcare systems.

## Advancement to knowledge:

The present study contributes evidence regarding the effectiveness of guided imagery as a non-pharmacological intervention for reducing anxiety among pregnant working women, a population that has received limited attention in maternal mental health research. The findings support the integration of guided imagery into antenatal care and workplace wellness programs to improve maternal psychological well-being.

## Conclusion:

We show that a four-week guided imagery intervention significantly reduced anxiety among pregnant working women, supporting its effectiveness as a non-pharmacological approach for antenatal anxiety management. Guided imagery is a safe, simple and cost-effective technique that can be integrated into antenatal care and workplace wellness programs. Further research with larger samples and randomized controlled designs is recommended to confirm its long-term effectiveness and generalizability.

## Competing Interests: 

Author declares no conflict of interest.

## Funding:

No specific grant from private, public, nonprofit funding organization has been obtained for the present study.

## Figures and Tables

**Table 1:** Frequencies and percentages were computed to describe the sample characteristics (N=50)

**CHARACTERISTICS**	**N**	**%**
**Age (IN YEARS)**		
20-25	4	8
26-30	7	14
31-35	20	40
36-40	19	38
**Education**		
10th Class	7	14
12th Class	6	12
Graduation	20	40
Post-Graduation & above	17	34
**Marital Status**		
Married	24	48
Separated	11	22
Divorced	11	22
Widowed	4	8
**Types of Family**		
Joint Family	31	62
Nuclear Family	19	38
Monthly income		
< Rs.30,000	21	42
Rs.30,001- Rs. 50,000	16	32
Rs.50,001- Rs. 70,000	8	16
> Rs.70,000	5	10
**Gestational weeks**		
First trimester - Conception to 12 weeks	20	40
Second trimester - 12 to 24 weeks	10	20
Third trimester - 24 to 35 weeks	20	40
**Gravida**		
Primi gravida	31	62
Multi gravida	19	38
Occupation type		
Government Job	20	40
Private Job	20	40
Self-employed	10	20
**Working hours per day**		
6-8 hours	20	40
More than 8 hours	30	60

**Table 2:** Distribution of pre-test and post-test anxiety levels among pregnant working women (N = 50)

**Anxiety Level**	**Pre-Test Frequency**	**Pre-Test Percentage (%)**	**Post-Test Frequency**	**Post-Test Percentage (%)**
Minimal Anxiety	5	10	22	44
Mild Anxiety	14	28	18	36
Moderate Anxiety	22	44	8	16
Severe Anxiety	9	18	2	4
Total	50	100	50	100

**Table 3:** Comparison of pre-test and post-test anxiety scores (paired t-test) (N = 50)

**Tests**	**Mean**	**SD**	**Mean Difference**	**t Value**	**P Value**
Pre-Test Anxiety Score	24.36	5.12	8.28	9.74	<0.001*
Post-Test Anxiety Score	16.08	4.37			
*Significant at p < 0.05

**Table 4:** Association between Post-test anxiety levels and selected demographic variables (N=50)

**CHARACTERISTICS**	**Stress Score (Post Test)**				**Df**	** χ^2^ Value**	**P Value**
	**Minimal anxiety**	**Mild anxiety**	**Moderate Anxiety**	**Severe Anxiety**			
**Age (IN YEARS)**							
20-25	2	1	1	0	9	1.08	0.999
26-30	3	3	1	0			
31-35	9	7	3	1			
36-40	8	7	3	1			
**Education**							
10th Class	2	3	1	1	9	5	0.834
12th Class	2	3	1	0			
Graduation	8	8	3	1			
Post-Graduation & above	10	5	2	0			
**Marital Status**							
Married	10	9	4	1	9	2.48	0.981
Separated	4	4	2	1			
Divorced	4	4	2	1			
Widowed	1	1	1	1			
**Types of Family**							
Joint Family	18	9	3	1	3	6.93	0.074
Nuclear Family	4	9	5	1			
Monthly income							
< Rs.30,000	7	8	5	1	9	5.01	0.833
Rs.30,001- Rs.50,000	7	6	2	1			
Rs.50,001- Rs.70,000	4	3	1	0			
>Rs.70,000	4	1	0	0			
**Gestational weeks**							
First trimester - Conception to 12 weeks	8	7	4	1	6	1.58	0.954
Second trimester - 12 to 24 weeks	4	4	2	0			
Third trimester - 24 to 35 weeks	10	7	2	1			
**Gravida**							
Primi gravida	13	11	6	1	3	0.78	0.854
Multi gravida	9	7	2	1			
Occupation type							
Government Job	12	6	2	0	6	7.53	0.275
Private Job	6	7	5	2			
Self -employed	4	5	1	0			
**Working hours per day**							
6-8 hours	14	4	2	0	3	9.57	0.023
More than 8 hours	8	14	6	2			
